# Focused ultrasound neuromodulation on a multiwell MEA

**DOI:** 10.1186/s42234-021-00083-7

**Published:** 2022-01-27

**Authors:** Marta Saccher, Shinnosuke Kawasaki, Martina Proietti Onori, Geeske M. van Woerden, Vasiliki Giagka, Ronald Dekker

**Affiliations:** 1 Department of Microelectronics, Delft University of Technology, Delft, Netherlands; 2 Department of Neuroscience, Erasmus MC, Rotterdam, Netherlands; 3 Department of Clinical Genetics, Erasmus MC, Rotterdam, Netherlands; 4 Fraunhofer Institute for Reliability and Microintegration IZM, Berlin, Germany; 5 Philips Research, Eindhoven, Netherlands

**Keywords:** Ultrasound, Neuromodulation, Bioelectronics, Multielectrode arrays, Focused ultrasound

## Abstract

**Background:**

Microelectrode arrays (MEA) enable the measurement and stimulation of the electrical activity of cultured cells. The integration of other neuromodulation methods will significantly enhance the application range of MEAs to study their effects on neurons. A neuromodulation method that is recently gaining more attention is focused ultrasound neuromodulation (FUS), which has the potential to treat neurological disorders reversibly and precisely.

**Methods:**

In this work, we present the integration of a focused ultrasound delivery system with a multiwell MEA plate.

**Results:**

The ultrasound delivery system was characterised by ultrasound pressure measurements, and the integration with the MEA plate was modelled with finite-element simulations of acoustic field parameters. The results of the simulations were validated with experimental visualisation of the ultrasound field with Schlieren imaging. In addition, the system was tested on a murine primary hippocampal neuron culture, showing that ultrasound can influence the activity of the neurons.

**Conclusions:**

Our system was demonstrated to be suitable for studying the effect of focused ultrasound on neuronal cultures. The system allows reproducible experiments across the wells due to its robustness and simplicity of operation.

**Supplementary Information:**

The online version contains supplementary material available at (10.1186/s42234-021-00083-7).

## Background

In the past decades, several methods for treating neurological disorders such as epilepsy, Parkinson’s disease and motor neuron disorders have been developed as an alternative to drugs. The most common method is to use electrical stimulation, however, other stimulation methods are recently considered. Among them, Low Intensity Focused Ultrasound (LIFU) is gaining particular attention because it can be focused in the submillimetre range, and its effects are reversible (Naor et al. 2016). Although its neuromodulation capabilities were observed almost one century ago (Harvey 1929), the scientific community has not agreed yet on the exact mechanisms responsible for the excitation or inhibition of the neurons with LIFU, and it is considered most likely that different mechanisms dominate depending on the sonication frequency (Kamimura et al. 2020). A broad range of ultrasound (US) parameters have been tested, however a gap in the research on the effects of high frequency neuromodulation exist, especially concerning which combination of parameters exactly causes excitation or inhibition of the neurons (Blackmore et al. 2019). Most of the studies on ultrasound neuromodulation have been done in vitro and on small animals, while only a few studies have been done on humans. Experiments on in vivo animals are complex due to the handling of the animals and the influence of anaesthesia on the response to ultrasound (Kamimura et al. 2020; Jerusalem et al. 2019). A simpler system to study the effect of ultrasound neuromodulation is to use cultured neurons on a microelectrode array (MEA). This makes it possible to study the effect of different ultrasound parameters on the activity of neurons, as well as using drugs to characterize the mechanism underlying these effects. Furthermore, significant advancements in human stem cell technology have enabled the quick and efficient generation of human neurons in a dish that could be used to test potential ultrasound treatments to find the optimal solution for a specific patient (Keller and Frega 2019).

Current MEA systems allow both electrical stimulation and recording of the cell activity, and integrating other neuromodulation methods will greatly increase the versatility of the MEA platform. Although other studies have tested the effects of ultrasound on in vitro neuronal cultures (Rinaldi et al. 1991; Tyler et al. 2008; Lin et al. 2018; Prieto et al. 2018; Kim et al. 2016; Hee-Sok et al. 2014), most of them used frequencies below 1MHz. Here, we describe the integration of a 24-well MEA system with an ultrasound delivery system that allows the testing of different wells during the same experiment.

## Methods

### Mice

FvB/NHsD females were crossed with FvB/NHsD males (ordered at 8–10 weeks old from Envigo) for the neuronal cultures. Mice were kept group-housed in IVC cages (Sealsafe 1145T, Tecniplast) with bedding material (Lignocel BK 8/15 from Rettenmayer) on a 12/12 h light/dark cycle in 21 ^∘^*C*(±1)^∘^C, humidity at 40-70%. Food pellets (801727CRM(P) from Special Dietary Service) and water were available ad libitum. All animal experiments were conducted in accordance with the European Commission Council Directive 2010/63/EU (CCD approval AVD101002017893).

### Primary hippocampal culture preparation and plating on the mEA

Primary hippocampal cultures were prepared from FvB/NHsD wild-type mice according to the procedure described by Goslin and Banker (Banker and Goslin 1991). Hippocampi were isolated from brains of E16.5 embryos and collected in 10 ml of neurobasal medium (NB, Gibco) on ice. After two washings with NB, the samples were incubated in pre-warmed trypsin/EDTA solution (Invitrogen) at 37 ^∘^C for 20 min. After two washings in pre-warmed NB, the cells were resuspended in 500 uL NB medium supplemented with 2% B27, 1% penicillin/streptomycin, and 1% glutamax (Invitrogen), and dissociated using a 1 ml pipette. Following dissociation, neurons were plated in a small drop in the centre of each well of a 24-wells MEA plate precoated with poly-d-lysine (25mg/ml, Sigma) at a density of 35000 cells per well. The plates were stored at 37 ^∘^C/5% CO_2_ for 45 minutes and 500 ul of pre-warmed supplemented NB medium was later added to each well. One-third of the medium was consecutively refreshed every three days.

### MEA and acquisition system

**Fig. 1:**
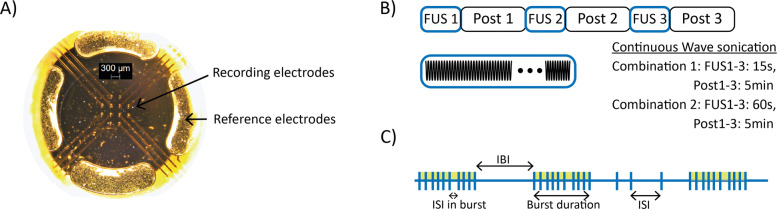
Experiment features. **A**) Microscope view of MEA electrodes. In the centre the 12 recording electrodes and at the sides, the 4 reference electrodes. **B**) Sonication protocol. **C**) Spike train features

The MEA plates used in this work have 24 wells, each with 12 gold recording electrodes and 4 reference electrodes coated with PEDOT (poly3,4-ethylene-dioxythiophene). Each recording electrode has a diameter of 30 um, and it is arranged on a 4x4 grid at a pitch of 300 um (MultiChannelSystem, Germany). The electrodes layout is illustrated in Fig. 1A. The MEA plates are coupled to the MEA readout headstage (MultiChannelSystem, Germany), which connects the recording electrodes to a computer running the acquisition software. The extracellular signals were recorded at a sampling frequency of 10 kHz and filtered using a 4^*th*^-order low-pass Butterworth filter with a cut-off frequency of 2 kHz and a 2^*nd*^-order high-pass Butterworth filter with cut-off frequency of 100 Hz. The baseline activity of the neurons was recorded for 5 minutes previous to the start of the ultrasound exposure for each well used in the experiment.

### System design

**Fig. 2:**
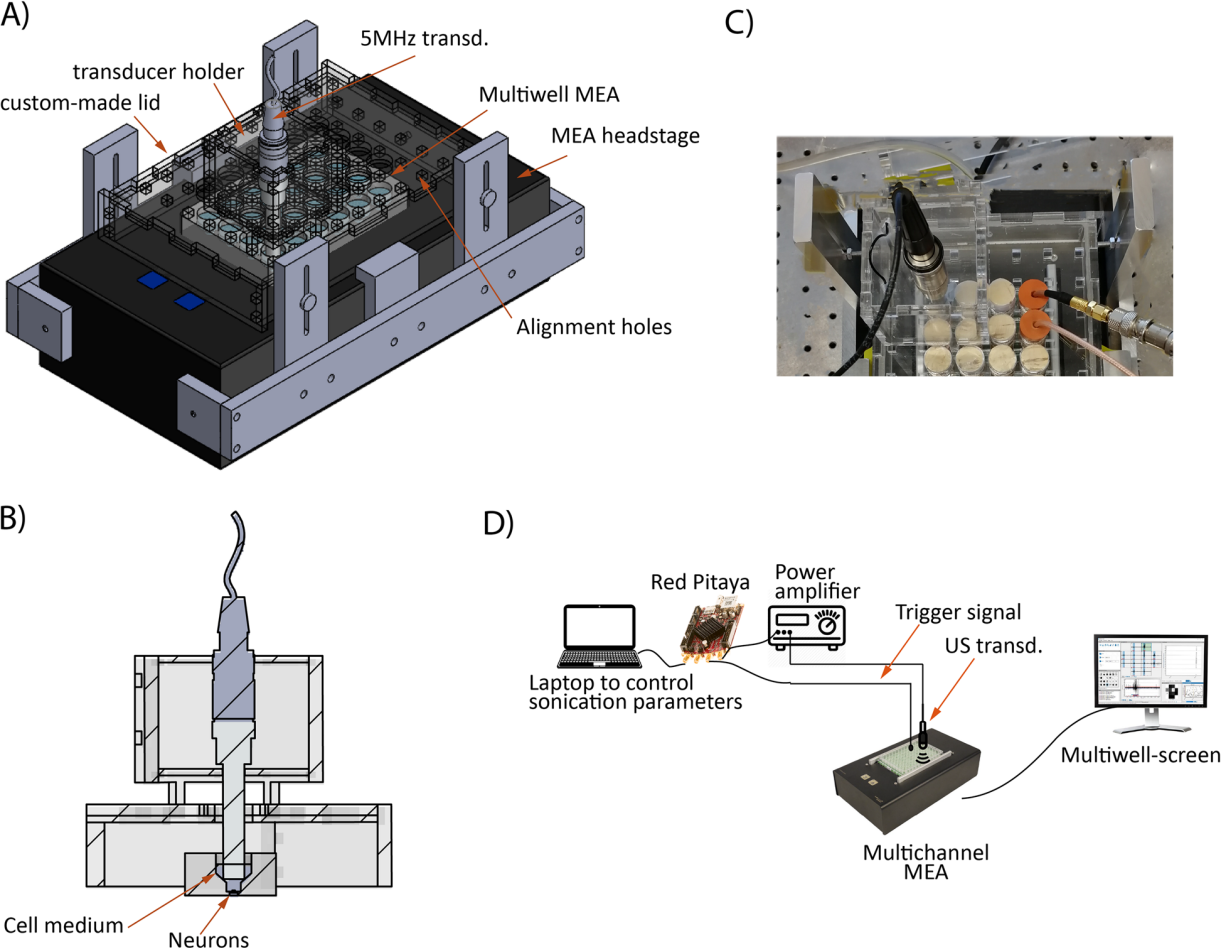
Experimental setup. **A**) 3D model of the experimental setup. The black box at the bottom is the MEA headstage, at the top of which the custom-made lid is placed. The circular holes in the lid are aligned to the underlying MEA plate. The ultrasound transducer is inserted in the transducer holder structure which can then be positioned in the engraved alignment holes for precise centering with respect to the MEA well. **B**) Cross-section view of the experimental setup showing the relative position of the transducer with respect to the neuronal culture. **C**) Detail picture of the experimental setup showing the transducer in the support structure on the left and on the right, the wells with the coloured cap are used to record the trigger signal. **D**) Schematic view of the complete experimental setup showing the connections between the various elements

A custom lid was designed and fabricated to cover the MEA headstage to ensure constant ambient conditions over the MEA plate of 5% CO_2_ and 95% O_2_ using an external oxygenation system interfaced to the lid via a small tube. Ultrasound was generated by a focused piezoelectric ultrasound transducer (V310, Olympus Panametrics NTD). The transducer is a single element curved transducer with a centre frequency of 5 MHz. It is spherically focused at 10 mm, with a focal length of 11 mm and a beam diameter at the focus point of approximately 600 um (-6dB). The ultrasound transducer has a cylindrical shape, therefore circular holes of the same diameter of the transducer were cut in the lid concentric to each MEA well to allow the US transducer to enter the well. A rigid mechanical support structure for the US transducers was designed to ensure a constant distance and alignment between the bottom of the transducer and the surface of the well plate. Alignment holes were engraved onto the lid to allow the transducer support structure to be easily and precisely positioned with respect to the lid and the MEA well (Fig. 2). In addition, few drops of water were placed on the tip of the transducer and then sealed with Parafilm^®^M film. This ensured the coupling of the transducer to the cell medium and prevented contamination of the transducer with biological material. In addition, the transducer was electrically isolated from the recording electrodes. Holes corresponding to unused wells were capped to maintain the ambient conditions.

### Program for generation of the sonication protocol

A RedPitaya board (RedPitaya) was used to generate the sinusoidal signal for the ultrasound transducer. This small board contains two programmable function generators that can be programmed to generate arbitrary waveform patterns. The board was connected to a power amplifier (240L, Electronics & Innovation Ltd.) The output of the power amplifier was, in turn, connected to the ultrasound transducer. The RedPitaya board also generated a trigger signal to indicate the ON- and OFF-time of the ultrasound signal. The electrodes of one well of the MEA system were used to record the trigger signal to provide the time stamp needed to correlate the neuron response to the US stimulation. The LED indicators on the RedPitaya board were also programmed to indicate that the ultrasound was ON and to signal the time left to the end of the trial. The complete experimental setup is depicted in Fig. 2D.

### Sonication protocol

Two different sonication protocols were chosen for the validation of the FUS system on the multiwell-MEA. Continuous ultrasound at a frequency of 5 MHz and exposure times of 15 and 60 seconds were used. These treatment durations were chosen based on the range of values found in the literature for continuous wave neuromodulation (Blackmore et al. 2019). Each treatment was repeated three times with an observation period, namely the post period, of 5 minutes between each treatment to test the consistency of the response to the ultrasound neuromodulation and the reversibility of this potential treatment method. The experiment was performed twice for each sonication protocol (Fig. 1B).

### Data analysis

Extracellular signals recordings were obtained using the Multiwell-Screen software application and analysed using the Multiwell-Analyzer software, both from MultiChannelSystem. The threshold for spike detection was set to ±15uV. Bursts were identified when at least 5 consecutive spikes were detected at an interval of less than 50 ms, defined as the inter-spike interval in burst (ISI in burst). The minimum interval between consecutive bursts was set to 100 ms, defined as inter-burst interval (IBI) (Chiappalone et al. 2005) (Fig. 1C). Data from the electrodes in each well was averaged to consider each well as a whole, in each different phase of the experiment. Data was then normalised with respect to the baseline activity, recorded previously to the exposure to ultrasound. Parameter calculation and analysis were done using custom MATLAB scripts, and results from different wells, in which the same ultrasound treatment duration was used, were then compared.

## Results

### System characterisation

#### Pressure measurements

Direct measurement of the acoustic pressure at the bottom of the well is not possible in this setup. Therefore, a separate measurement was performed in a water tank setup using a calibrated hydrophone needle (Precision Acoustic Ltd.) mounted on a high precision x-y-z scanner. The input voltage of the ultrasound transducer was the same as the one used during the cell experiments. The hydrophone needle was aligned to the centre of the transducer and scanned in a plane parallel to the face of the transducer, at the same distance as between the bottom of the well and the transducer in the cell experiments. An area equivalent to the bottom of the MEA well was mapped, and for each location, the peak-to-peak pressure values were calculated by dividing the peak-to-peak values of the recorded signal by the sensitivity of the needle at the corresponding frequency.

#### Modelling of ultrasound field

**Fig. 3:**
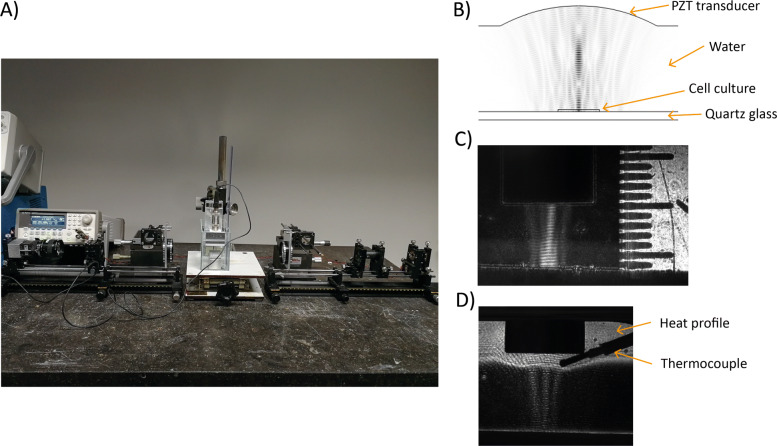
Schlieren setup. **A**) View of the complete setup. **B**) Result of ultrasound field simulation in COMSOL. **C**) Interference pattern generated by the reflection of the ultrasound field on a hard surface. The distance between the ultrasound transducer and the reflecting surface is the same as the distance between the transducer and the bottom of the MEA well (6 mm). **D**) Heat profile generated by the ultrasound transducer at intensity 4 times higher than the experimental conditions. The shape of the thermocouple is also visible

To estimate the effect of the MEA plate on the ultrasound field, a finite element model was computed in COMSOL (Multiphysics). A simulation consisting of the ultrasound transducer immersed in an infinite water medium was computed and matched to the hydrophone pressure measurements. The structure of the MEA well was then included in the model, which was analysed using pressure acoustic and solid mechanics models. The bottom of the MEA well was assumed to be entirely made of quartz glass and the smaller structures, like electrodes, were not included in the model because their size is one tenth of the wavelength, therefore negligible. The thickness of the quartz glass was 600 um, and it was modelled as a perfectly reflecting surface with respect to the ultrasound waves. The culture medium was approximated as water at 37 ^∘^C, and the primary hippocampal neurons layer was assumed to be an incompressible and hyperplastic material with a thickness of 150 um (Menz et al. 2019). The distance between the ultrasound transducer face and the bottom of the plate was 6 mm. A symmetrical boundary condition was applied due to the radial symmetry of the geometry. A frequency-domain study was chosen because the system is actuated at a harmonic frequency of 5 MHz. The acoustic properties of the primary hippocampal neurons were retrieved from the literature (IT’IS 2021; Lay et al. 2019) and are summarised in Table 1, while the COMSOL default values for the properties of the other materials in the model were used. To solve the model, a free triangular mesh with a size of *λ*/8 was chosen, and the default solver was used. In Fig. 3B, the results of the pressure acoustic study show the presence of an interference pattern generated by the reflections of the acoustic waves at the bottom of the MEA well. In addition, due to the presence of the glass layer at the bottom of the MEA well, the maximum pressure at the level of the neurons was simulated to be 1.05MPa.

**Table 1:** COMSOL parameters used for the hippocampal neurons

Parameter	Value
Speed of sound	1500 [m/s]
Density	1045 [kg/m ^3^]
Thermal conductivity	0.55 [W/m/K]
Heat capacity	3696 [J/kg/K]
Shear modulus	2 [kPa]

#### Validation of the computed ultrasound field

To validate the results of the COMSOL simulation of pressure field, a pulse laser Schlieren system was used (Fig. 3A). The Schlieren system is an optic system that allows the ultrasound field to be visualised, being able to image the change in the refractive index of a transparent medium (Neumann and Ermert 2006). For this work, the ultrasound transducer was immersed in a glass bath filled with water. The distance between the tip of the transducer and the bottom of the water bath was kept the same as the distance between the transducer and the bottom of the MEA well. The camera of the Schlieren system visualises the ultrasound waves as they are moving through the water. In this way, it is also possible to follow the temporal evolution of the ultrasound field. The ultrasound transducer was driven with the same input voltage used during the cells experiment, and in Fig. 3C, the interference pattern generated by the reflections of the ultrasound beam on the bottom of the water bath in which the transducer is submerged is shown. Comparing Fig. 3B and C, the similarity between the COMSOL simulated field and the one pictured with the Schlieren setup is evident. Nevertheless, as reported in (Andrews 2014), the exact interpretation of Schlieren images is rather difficult and should be considered as a validation and illustration of the results from the FEM simulations.

#### Validation of the temperature increase

To assess the increase in temperature at the location of the cells during the experiments, a K type thermocouple with a sensitivity of ±0.1^∘^C was placed at the bottom of one of the wells of the multiwell MEA plate. The well was filled with water and placed on a temperature-controlled surface, at 36 ^∘^C. The ultrasound transducer was then placed inside the well and turned ON for 60 seconds with the same input voltage as during the cell experiments. This resulted in a maximum temperature increase of 0.8 ^∘^C. The temperature returned to the starting value within 5 minutes after the ultrasound was turned OFF. In addition, the Schlieren system can also be used to visualise heat profiles since temperature differences reflect in different densities of the medium, hence different refractive indexes. The ultrasound transducer was placed in the same Schlieren setup configuration used to validate the ultrasound field, and the same thermocouple as the one used for the first experiment was placed close to the tip of the transducer. The ultrasound transducer was then turned ON for 60 seconds with the same input voltage as during the cell experiments. However, due to the small temperature increase, the heat profile was difficult to visualise due to the resolution limits of the Schlieren system, therefore a third experiment was performed driving the ultrasound transducer with an input voltage 4.5 times higher than the one used during the cell experiments, to better visualise the heat profile. Figure 3D depicts the resulting heat profile at 60 seconds. The maximum increase in temperature was of 3.3^∘^C. In the last two experiments, the objective was to assess the heat profile and not the actual temperature rise, since the liquid volume of the bath used in the Schlieren system is much larger than the one of the MEA well. Moreover, it can be noticed that the heat profile is shallow to the tip of the transducer and the water surface, suggesting that, even at a much higher intensity, the heat does not reach the neuronal culture and is dispersed at the liquid surface rather than towards the neurons. The video recordings associated with these experiments are publicly available at (Saccher et al. 2021).

### Validation of the fUS system for neuromodulation on multiwell mEA

**Fig. 4:**
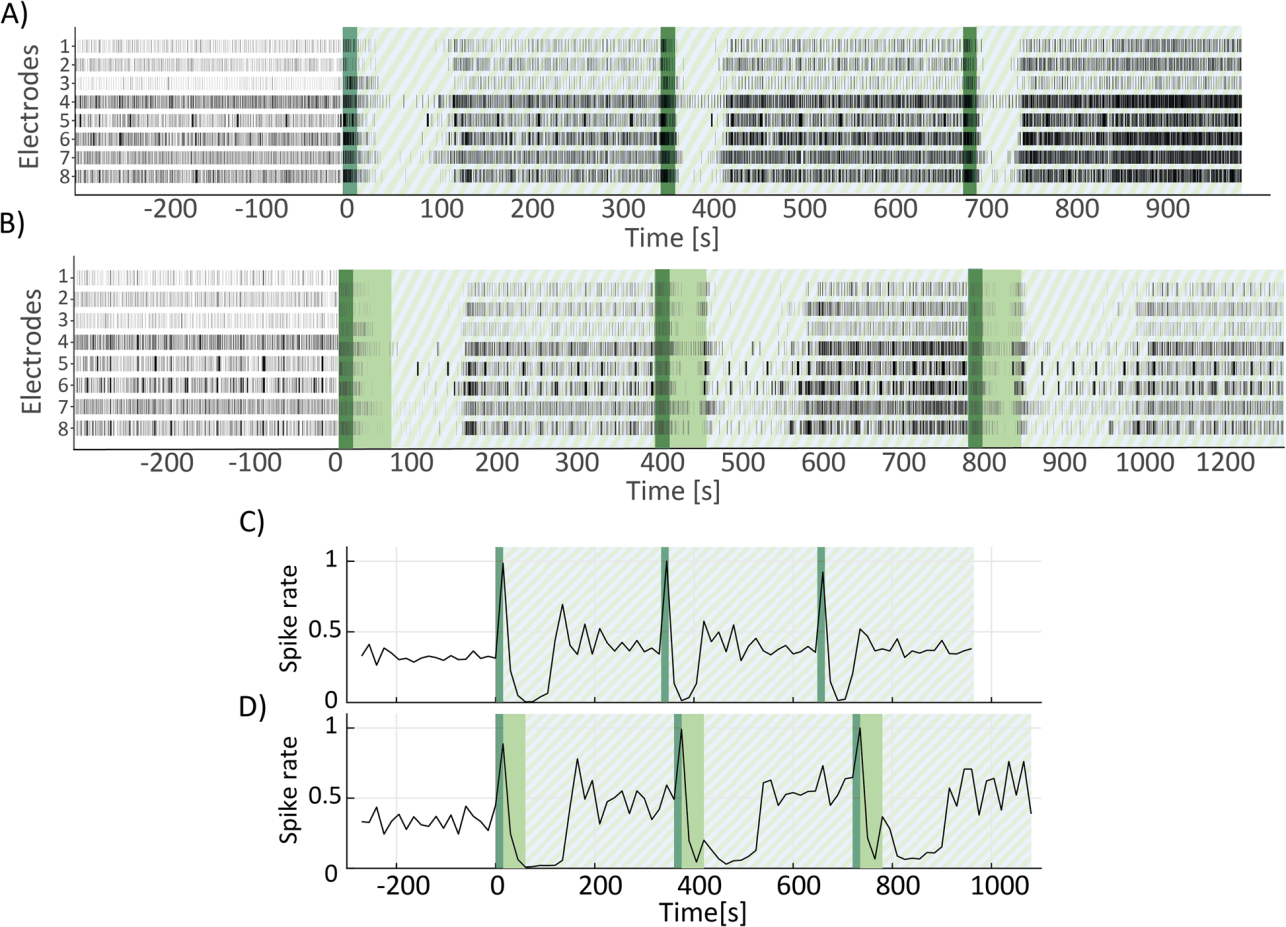
Ultrasound neuromodulation results. A) Example of raster plot of one of the wells used in the experiment, for a treatment duration of 15 seconds. Dark green shaded area indicates the US-ON period, while stripes-patterned area indicates the US-OFF period, namely the post period. B) Same as A) but for a treatment duration of 60 seconds. Dark green shaded area indicates the first 15 seconds of US-ON period and light green area indicates the remaining 45 seconds. Stripes-patterned area indicates the US-OFF period. C) Spike rate trend corresponding to the raster plot in A), calculated for 15 seconds bins over the duration of the experiment. Dark green shaded area indicates the US-ON period and stripe-patterned area indicates the US-OFF period. D) Spike rate trend corresponding to the raster plot in B), calculated for 15 seconds bins over the duration of the experiment. Dark green shaded area indicates the first 15 seconds of US-ON and light green area the remaining 45 seconds. Stripe-patterned area indicates the period of US-OFF

The focused ultrasound system was tested on mouse primary hippocampal neurons. Preceding the exposure to ultrasound, activity of the neuronal culture was recorded for 5 minutes as a baseline measurement in seven MEA wells. During the periods of US-ON (15 second or 60 second period), neurons initially showed an increased firing rate, as shown in Fig. 4A and B, immediately followed by a reduction or even a pause in firing, after which the neurons resumed their baseline firing pattern. However, the response to the stimulus showed some differences in the two conditions. In the 15 second condition, the increased firing pattern was observed during the total stimulation time, followed by a pause in firing. In the 60 second condition, only in the first 15 seconds of the 60 second stimulus, the firing frequency was increased. In the subsequent 45 seconds stimulus, the firing frequency already showed a strong reduction (Fig. 4D). The duration of the post-stimulus pause appeared to last longer in the 60 second than in the 15 second condition (Fig. 4C and D), however a precise calculation of this variable was not possible due to the low *n* and the variability in the activity recorded by the electrodes. The responses of the other wells are presented in Supplemental Figure 1 & 2. These results indicate that the ultrasound stimulation can affect neuronal activity.

## Discussion

In this work, the use of a multiwell plate in combination with an ultrasound delivery system to study the influence of focused ultrasound on the neural activity was shown. Other studies performed in vitro ultrasound neuromodulation yet using a wide variety of experimental setups, mostly ignoring the presence of reflections and standing waves patterns. In FUS neuromodulation applications, reflections and standing waves are relevant because the actual pressure amplitude or intensity level to which the neurons are exposed is different from what can be measured in an acoustic measurement setup. This is because measuring the actual pressure field in the real experimental conditions is very difficult, if not impossible, since the tools used to perform these measurements cannot be used in vivo or easily integrated in in-vitro experiments. Mediums with different densities or mechanical properties such as bones, skin or other organs cause standing waves and reflections due to their different acoustic impedances. For example in transcranial FUS, reflections are created at the boundaries between the transducer and the skull, and the skull and the brain, especially at frequencies above 1 MHz (Pinton et al. 2011). In addition, ultrasound waves reflect off the opposite side of the skull creating standing waves and pressure hotspots in the brain at locations other than the target point (Younan et al. 2013). As a consequence, the actual ultrasound field at the target location is different from what could be measured in an experimental setup. The uncontrolled presence of standing waves in an experiment makes the comparison of the results from different studies unreliable, hence the need to more accurately measure and analyse the real experimental conditions, which, in this work, was done with finite element methods simulations and the use of Schlieren imaging.

The MEA plate and the ultrasound delivery system were integrated and characterised with pressure measurements and simulations of the experimental conditions. The simulations highlighted the presence of standing waves between the transducer and the bottom of the MEA well. This is due to the impedance mismatch between the cell medium and the two hard surfaces, namely the quartz glass and the ultrasound transducer, making the acoustic wave reflect back and forth between them. The resulting pattern depends on the distance between the two elements. Locations at *λ*/2 experience constructive interference (nodes) while locations at *λ* experience destructive interference (antinodes), with *λ* the acoustic wavelength. Standing waves increased the pressure inside the MEA well, compared to the pressure measured with the hydrophone at the same location and in a water tank. This was also observed in another study, (Secomski et al. 2017) and it could be potentially used to reduce the intensity generated by the ultrasound transducer.

Acoustic pressure is converted into radiation pressure due to absorption. This generates a pattern of stretching and compression at the location of the nodes and antinodes, which can influence some ionic channels or the membrane capacitance, with an impact on the neural activity. In addition to mechanical effects, heat is also a possible neuromodulation mechanism that is able to affect the spiking rate, amplitude and duration of action potentials. However, previous studies suggest that for temperature variations of less than a degree, the effect on neuromodulation is negligible (Tyler et al. 2008; Kim et al. 2019; Khraiche et al. 2008; Tufail et al. 2010), while a temperature increase of a few degrees was found to cause a decrease in spiking activity (Schiff and Somjen 1985; Gulick et al. 2017). In our experiments, the results of the measurements with a thermocouple indicate that the temperature increase was 0.8 ^∘^C, and that the temperature resettled to the initial value within 5 minutes, hence during the recovery time of the neuromodulation treatment. In addition, the measurement of the heat profile with the Schlieren system suggests that most of the heat does not reach the level of the neuronal culture excluding thermal damage and thermal effects as potential mechanisms for neuromodulation.

The functioning of the newly designed system was validated with a proof-of-concept experiment on primary hippocampal neurons, showing that ultrasound affected the activity of the neurons. It was observed that the neuronal spiking activity increased during the period of US-ON. These results are comparable to what was observed by (Khraiche et al. 2008) using ultrasound at 7.75 MHz at an intensity higher than 50 W/cm ^2^. A period of reduced spiking activity followed each ultrasound treatment, which could be due to a feedback mechanism from interneurons, also present in the cultures. This phenomenon (of reduced firing) can also be observed after electrical stimulation of the neurons, although the duration of the electrical stimuli is usually 3 orders of magnitude lower than the ones used for ultrasound neuromodulation in this experiment (Hales et al. 2010). The response of the hippocampal neurons was consistent across three consecutive treatments and, in the recovery period following each ultrasound treatment, the spiking activity tended to values similar to the baseline, as observed in other studies (Hee-Sok et al. 2014; Choi et al. 2013). However, the strength of the response differed when looking at different wells (Supplemental Figure 1 & 2). What exactly causes this difference in response is difficult to say based on the low *n*. However, the baseline firing pattern of the neurons differed across the wells, which could be due to, amongst others, the density of the neurons plated in the dish and/or the number of dead cells. This could potentially be an explanation for the variation in responses to FUS. Future experiments will investigate how differences in cell densities and other biological factors influence the responses seen with FUS.

Previous studies showed that FUS at 43 MHz can activate the mechanosensitive Piezo1, but not the voltage-gated sodium channel *Na*_*V*_1.2 (Prieto et al. 2018). Additionally, using the same FUS frequency on hippocampal slices, an effect was found on the resting membrane potential and the shape of the action potential of CA1 pyramidal neurons (Prieto et al. 2020), suggesting that FUS indeed is capable of altering neuronal characteristics. The setup presented in this work can be used in future studies to gain more insight into whether the effects of different stimulation frequencies and ultrasound parameters can differentially affect neuronal activity. Primary hippocampal cultures of different knock-out or knock-in mouse models can be used, or neurons can be transduced with different shRNAs to knock down specific channels or proteins involved in neuronal signalling. Finally, specific inhibitors or enhancers of specific channels can be added to disentangle the mechanism underlying the effect of FUS on neuronal firing.

The system was proven to be easy to assemble and operate, given the simplicity of the positioning of the ultrasound transducer in each well, which leaves small margins for alignment errors and ensures the reproducibility of the experimental conditions across the different wells. The system was also made free from biological contamination due to the presence of the Parafilm^®^M film that isolates the ultrasound transducer from the cell medium yet not influencing the ultrasound field. The Parafilm^®^M layer also electrically isolates the recording electrodes from the ultrasound transducer, ensuring that the response of the neurons is influenced only by the ultrasound field.

## Conclusions

In this work, the integration of a focused ultrasound delivery system with a multiwell MEA plate was presented. The system was characterised by pressure measurements, Schlieren imaging and finite element methods simulations. This method could be used to correct and estimate the effects of a certain setup geometry and experimental conditions to find the optimal conditions for an experiment. A proof-of-concept experiment was performed on a primary hippocampal neurons culture, demonstrating the potential of this system for investigations of focused ultrasound neuromodulation. Future work will focus on studying the effects of ultrasound on neurons using different sets of parameters and higher ultrasound frequencies. The focusing effect of ultrasound on the response of the neurons will also be investigated, and future experiments will also explore the effects on different types of cells.

## Supplementary Information

**Additional file 1** Additional results of ultrasound neuromodulation. A) Raster plot of a well with a treatment duration of 15 seconds. Dark green shaded area indicates the US-ON period, while stripes-patterned area indicates the US-OFF period, namely the post period. B) Spike rate trend corresponding to the raster plot in A), calculated for 15 seconds bins over the duration of the experiment. Dark green shaded area indicates the US-ON period and stripe-patterned area indicates the US-OFF period. C,E) Same as A) but for a treatment duration of 60 seconds. Dark green shaded area indicates the first 15 seconds of US-ON period and light green area indicates the remaining 45 seconds. Stripes-patterned area indicates the US-OFF period. D,F) Spike rate trend corresponding to the raster plot in C) and E) respectively, calculated for 15 seconds bins over the duration of the experiment. Dark green shaded area indicates the first 15 seconds of US-ON and light green area the remaining 45 seconds. Stripe-patterned area indicates the period of US-OFF.

**Additional file 2** Additional results of ultrasound neuromodulation. A) Raster plot of a well with a treatment duration of 15 seconds. Dark green shaded area indicates the US-ON period, while stripes-patterned area indicates the US-OFF period, namely the post period. B) Spike rate trend corresponding to the raster plot in A), calculated for 15 seconds bins over the duration of the experiment. Dark green shaded area indicates the US-ON period and stripe-patterned area indicates the US-OFF period. C) Same as A) but for a treatment duration of 60 seconds. Dark green shaded area indicates the first 15 seconds of US-ON period and light green area indicates the remaining 45 seconds. Stripes-patterned area indicates the US-OFF period. D) Spike rate trend corresponding to the raster plot in C), calculated for 15 seconds bins over the duration of the experiment. Dark green shaded area indicates the first 15 seconds of US-ON and light green area the remaining 45 seconds. Stripe-patterned area indicates the period of US-OFF.

## Data Availability

The dataset used during the current study is available from the corresponding author on reasonable request. The video recordings associated with the experiments are available in the *4TU.ResearchData* repository, 10.4121/17055329.v2.
