# Supplementary figures and images for: Focused ultrasound neuromodulation on a multiwell MEA
Source: Bioelectron Med. 2022 Jan 27;8:2. doi: 10.1186/s42234-021-00083-7 (PMC8793260; doi:10.1186/s42234-021-00083-7)

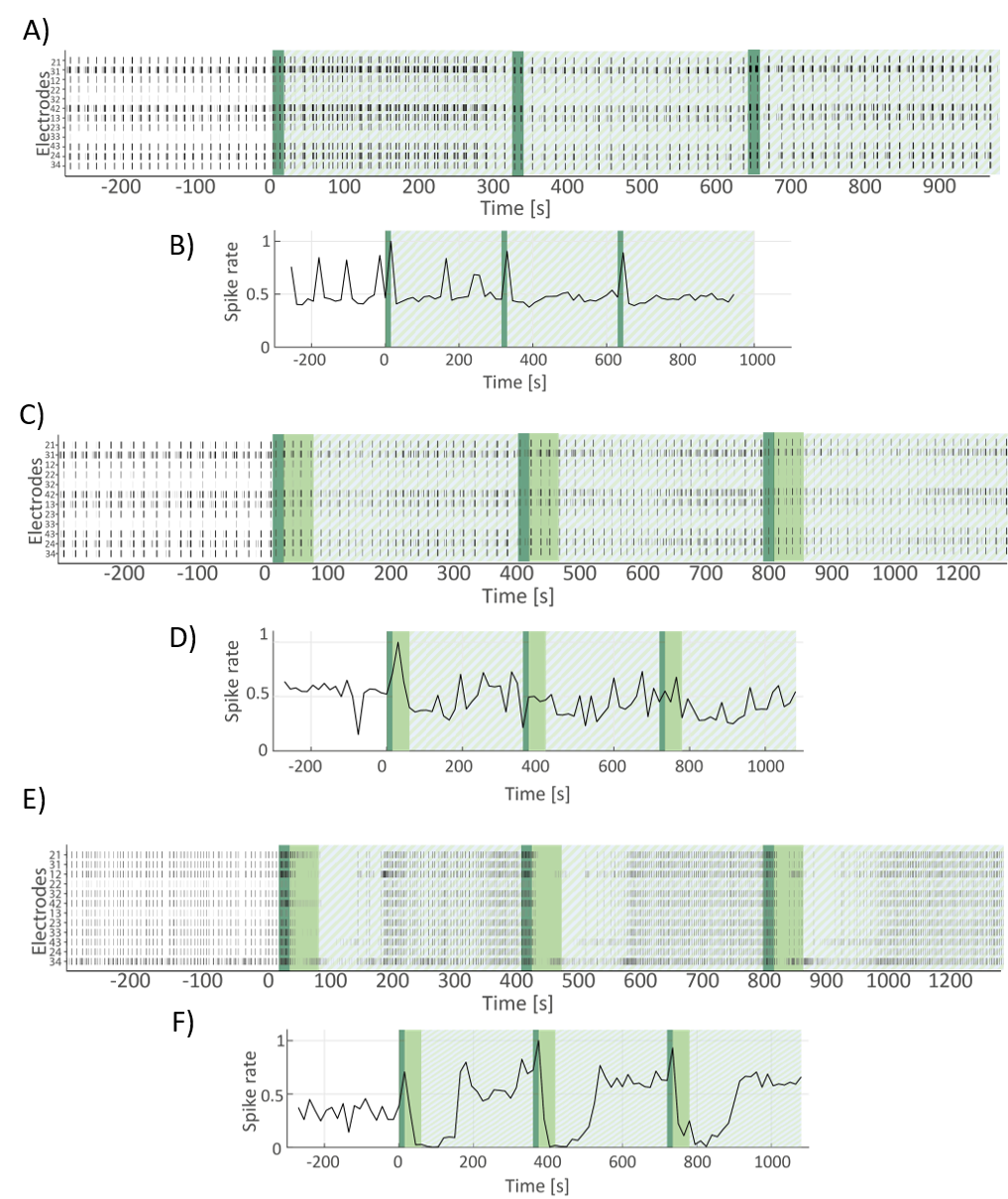

Supplement: Supplementary file 1 — Additional file 1 Additional results of ultrasound neuromodulation. A) Raster plot of a well with a treatment duration of 15 seconds. Dark green shaded area indicates the US-ON period, while stripes-patterned area indicates the US-OFF period, namely the post period. B) Spike rate trend corresponding to the raster plot in A), calculated for 15 seconds bins over the duration of the experiment. Dark green shaded area indicates the US-ON period and stripe-patterned area indicates the US-OFF period. C,E) Same as A) but for a treatment duration of 60 seconds. Dark green shaded area indicates the first 15 seconds of US-ON period and light green area indicates the remaining 45 seconds. Stripes-patterned area indicates the US-OFF period. D,F) Spike rate trend corresponding to the raster plot in C) and E) respectively, calculated for 15 seconds bins over the duration of the experiment. Dark green shaded area indicates the first 15 seconds of US-ON and light green area the remaining 45 seconds. Stripe-patterned area indicates the period of US-OFF. [file 42234_2021_83_MOESM1_ESM.png]

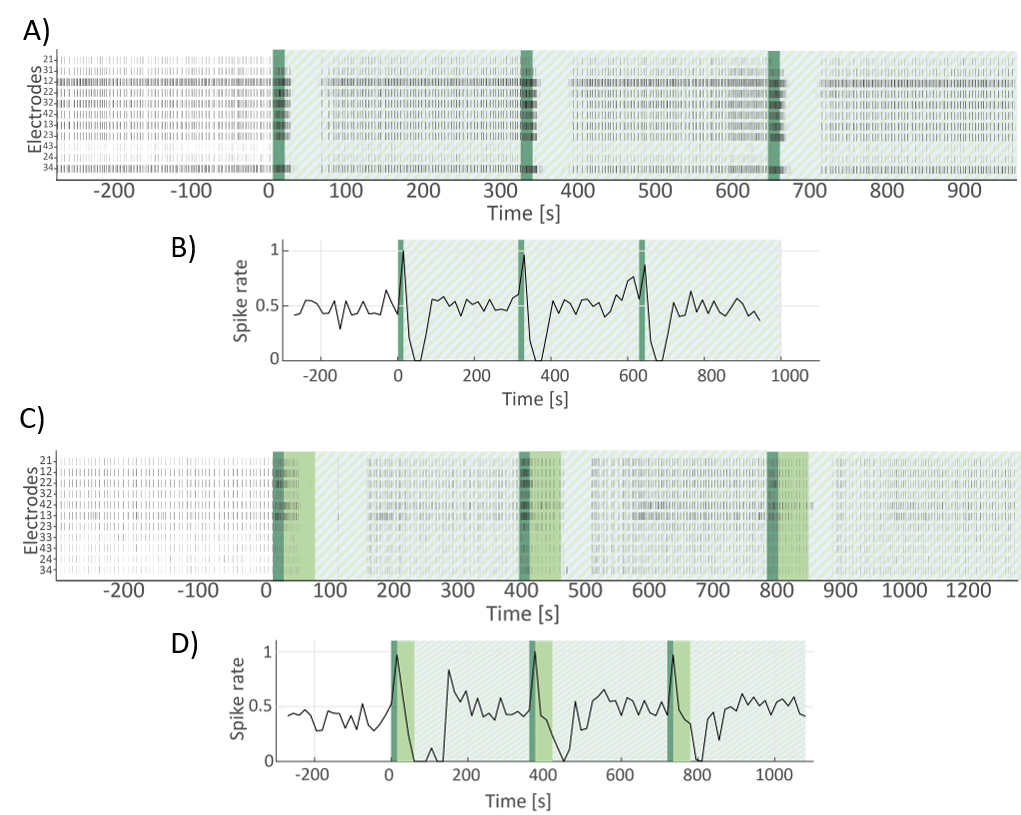

Supplement: Supplementary file 2 — Additional file 2 Additional results of ultrasound neuromodulation. A) Raster plot of a well with a treatment duration of 15 seconds. Dark green shaded area indicates the US-ON period, while stripes-patterned area indicates the US-OFF period, namely the post period. B) Spike rate trend corresponding to the raster plot in A), calculated for 15 seconds bins over the duration of the experiment. Dark green shaded area indicates the US-ON period and stripe-patterned area indicates the US-OFF period. C) Same as A) but for a treatment duration of 60 seconds. Dark green shaded area indicates the first 15 seconds of US-ON period and light green area indicates the remaining 45 seconds. Stripes-patterned area indicates the US-OFF period. D) Spike rate trend corresponding to the raster plot in C), calculated for 15 seconds bins over the duration of the experiment. Dark green shaded area indicates the first 15 seconds of US-ON and light green area the remaining 45 seconds. Stripe-patterned area indicates the period of US-OFF. [file 42234_2021_83_MOESM2_ESM.png]
